# Molecular mechanisms underlying the antitumor activity of (E)-N-hydroxy-3-(1-(4-methoxyphenylsulfonyl)-1,2,3,4-tetrahydroquinolin-6-yl)acrylamide in human colorectal cancer cells *in vitro* and *in vivo*

**DOI:** 10.18632/oncotarget.5475

**Published:** 2015-10-05

**Authors:** Chun-Han Chen, Chia-Hwa Lee, Jing-Ping Liou, Che-Ming Teng, Shiow-Lin Pan

**Affiliations:** ^1^ The Ph.D. program for Cancer Biology and Drug Discovery, College of Medical Science and Technology, Taipei Medical University, Taipei, Taiwan; ^2^ School of Pharmacy, College of Pharmacy, Taipei Medical University, Taipei, Taiwan; ^3^ Pharmacological Institute, College of Medicine, National Taiwan University, Taipei, Taiwan

**Keywords:** CRC, HDAC, apoptosis, EMT, Akt

## Abstract

Upregulation of class I histone deacetylases (HDAC) correlates with poor prognosis in colorectal cancer (CRC) patients. Previous study revealed that (E)-N-hydroxy-3-(1-(4-methoxyphenylsulfonyl)-1,2,3,4-tetrahydroquinolin-6-yl)acrylamide (Compound 11) is a potent and selective class I HDAC inhibitor, exhibited significant anti-proliferative activity in various human cancer cell lines. In current study, we demonstrated that compound 11 exhibited significant anti-proliferative and cytotoxic activity in CRC cells. Notably, compound 11 was less potent than SAHA in inhibiting HDAC6 as evident from the lower expression of acetyl-α-tubulin, suggesting higher selectivity for class I HDACs. Mechanistically, compound 11 induced cell-cycle arrest at the G2/M phase, activated both intrinsic- and extrinsic-apoptotic pathways, altered the expression of Bcl-2 family proteins and exerted a potent inhibitory effect on survival signals (p-Akt, p-ERK) in CRC cells. Moreover, we provide evidence that compound 11 suppressed motility, decreased mesenchymal markers (N-cadherin and vimentin) and increased epithelial marker (E-cadherin) through down-regulation of Akt. The anti-tumor activity and underlying molecular mechanisms of compound 11 were further confirmed using the HCT116 xenograft model *in vivo*. Our findings provide evidence of the significant anti-tumor activity of compound 11 in a preclinical model, supporting its potential as a novel therapeutic agent for CRC.

## INTRODUCTION

Histone deacetylase (HDAC) inhibitors represent a new emerging class of agents that induce a range of anticancer effects, including tumor cell apoptosis, cell cycle arrest, differentiation, senescence, modulation of immune responses, and altered angiogenesis [1]. Several lines of evidence indicate increased expression of class I HDACs (HDAC1, 2, 3, 8) in colorectal tumors relative to adjacent normal mucosa. This step may facilitate progression of tumors via transcriptional repression of genes that normally function in growth arrest, differentiation and apoptosis [2]. Accordingly, pharmacological inhibitors of HDACs have been experimentally utilized for the treatment of colorectal cancer. Several lines of evidence have suggested that combined treatments involving HDAC inhibitors plus tyrosine kinase inhibitors have synergistic effects in cancer cells [3–5]. Vorinostat (also known as SAHA; suberoylanilide hydroxamic acid), a pan-HDAC inhibitor, is currently used in the clinic to treat cutaneous T cell lymphoma (CTCL). In addition, a number of ongoing clinical trials have assessed the combined use of SAHA and erlotinib in patients with advanced NSCLC [6].

Genesis of the metastatic phenotype, which involves alterations of functions and structures in the epithelium, is the major cause of death in CRC patients [7]. These changes include dedifferentiation, loss of adhesive constraints and increased cellular motility, important hallmarks of epithelial-mesenchymal transition (EMT) [8]. EMT is considered a pathological process that contributes to cancer progression, particularly in relation to invasion, metastasis and drug resistance [9]. In CRC, it has been reported that tumor cells having undergone EMT are represented by the presence of tumor buds at the invasive front [10]. Proteins involved in transcriptional repression of *E-cadherin* gene, such as Snail, Slug and Twist, have been shown to contribute to invasion and metastasis in carcinoma progression [11]. Therefore, the EMT pathway presents a promising therapeutic target for developing new anti-cancer agents.

Compound 11 ((E)-N-hydroxy-3-(1-(4-methoxyphenylsulfonyl)-1,2,3,4-tetrahydroquinolin-6-yl)acrylamide) is a novel HDAC inhibitor with cytotoxicity in a variety of human cancer cell lines [12]. Of note, compound 11 is more potent than SAHA in lung cancer (A549) and CRC (HCT116) cells. In the present study, we examined the anti-cancer activity of compound 11 and its underlying mechanisms in human CRC cells. Our results revealed significant anti-proliferative and cytotoxic activity in CRC cells, and caspase-dependent activation of both intrinsic- and extrinsic-apoptotic pathways. Notably, compound 11 suppressed cell motility and reversed the mesenchymal phenotype through downregulation of Akt. Moreover, tumor growth in a HCT116 xenograft model was significantly suppressed by compound 11 *in vivo*. These findings collectively support the potential of compound 11 for further development as a therapeutic agent in CRC.

## RESULTS

### Compound 11 is a novel HDAC inhibitor with anti-proliferative and cytotoxic effects in colorectal cancer cells

**Figure 1:**
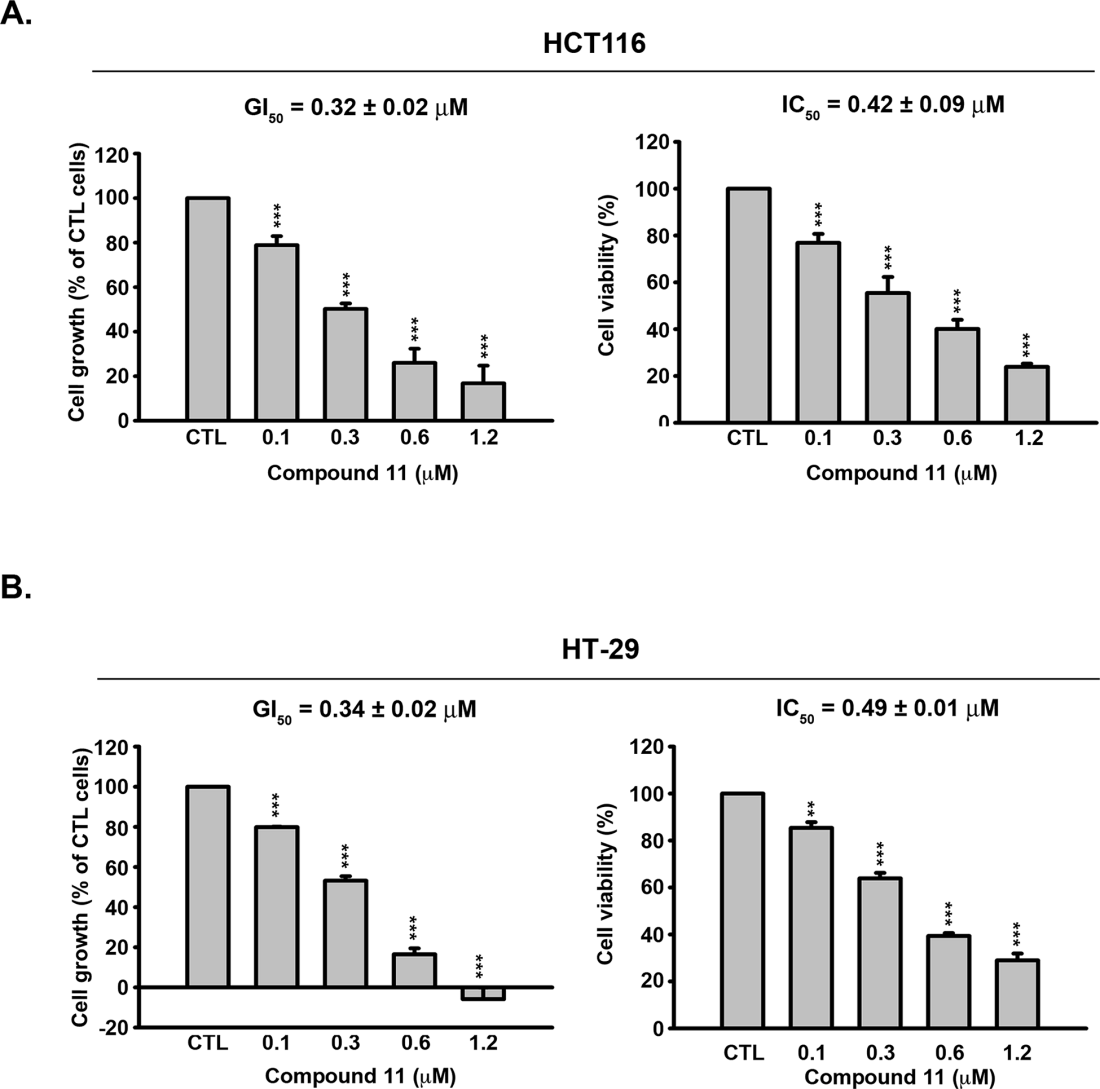
Effects of compound 11 on cell proliferation and viability in CRC cells Concentration-dependent effects of compound 11 on cell growth (left panel) and viability (right panel) of HCT116 **A.** and HT-29 **B.** cells. Cells were treated with DMSO or compound 11 at the indicated concentrations for 48 h, and cell growth and viability determined with the SRB and MTT assays, respectively. Data represent the mean values ± S.D. from three independent experiments. GI_50_ and IC_50_ are calculated as described in Materials and Methods. **P < 0.01; ***P < 0.001 compared with the control (CTL) group.

**Figure 2:**
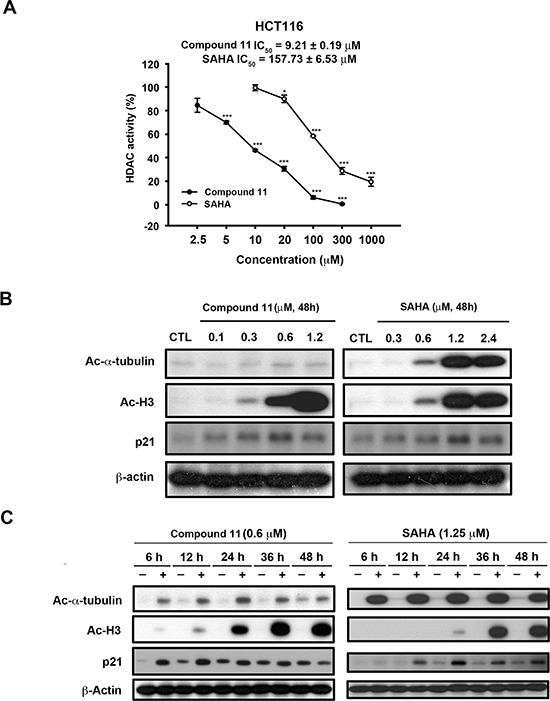
Effects of compound 11 on HDAC activity in HCT116 cells **A.** Comparison of the effects of compound 11 and SAHA on HDAC activity in HCT116 cells. After 24 h of drug treatment, whole cell lysates were collected and subjected to a fluorometric HDAC activity assay kit as described in Materials and Methods. **B–C.** Concentration- and time-dependent activities of compound 11 and SAHA on the expression of epigenetic markers (Ac-α-tubulin, Ac-H3 and p21). HCT116 cells were treated with compound 11 or SAHA at the indicated concentrations for 48 h (B) or a fixed concentration for different times (C) and whole cell extracts were harvested and subjected to Western blot analysis.

We initially examined the effects of compound 11 on proliferative activity and viability of human colon cancer cell lines, HCT116 (p53 wild-type, Figure 1A) and HT-29 (p53 mutant, Figure 1B). Compound 11 exerted significant anti-proliferative activity and cytotoxicity in both cell lines, despite their differing p53 status. In a previous study, we examined the target specificity of compound 11 using an *in vitro* HDAC inhibition assay. Compared with SAHA, compound 11 was 2- to 5-fold more potent against HDAC 1, 2, and 8, but is 8-fold less potent against HDAC 6 [12]. In the current study, the nuclear enzyme activity of compound 11 in HCT116 cell nuclear extracts was measured with the HDAC Fluorescent Activity Assay. Compound 11 exerted greater HDAC inhibition activity than SAHA in HCT116 cells with extrapolated IC_50_ value of 9.21 ± 0.19 μM, relative to 157.73 ± 6.53 μM for SAHA (Figure 2A). We further confirmed the epigenetic effects of compound 11 by analyzing the acetylation of histone and nonhistone proteins, and induction of the epigenetically silenced gene, p21. Exposure to compound 11 and SAHA led to upregulation of acetyl-histone H3, acetyl-α-tubulin, and p21 in a concentration- and time-dependent manner (Figure 2B and 2C). Notably, compound 11 was less potent than SAHA in inhibiting HDAC6 as evident from the lower expression of acetyl-α-tubulin, suggesting higher selectivity for class I HDACs. Our results provide evidence of the HDAC inhibitory activity of compound 11, which exerts anti-proliferative activity and cytotoxicity in colorectal cancer cells.

### Compound 11 induces cell cycle arrest and caspase-dependent apoptosis

**Figure 3:**
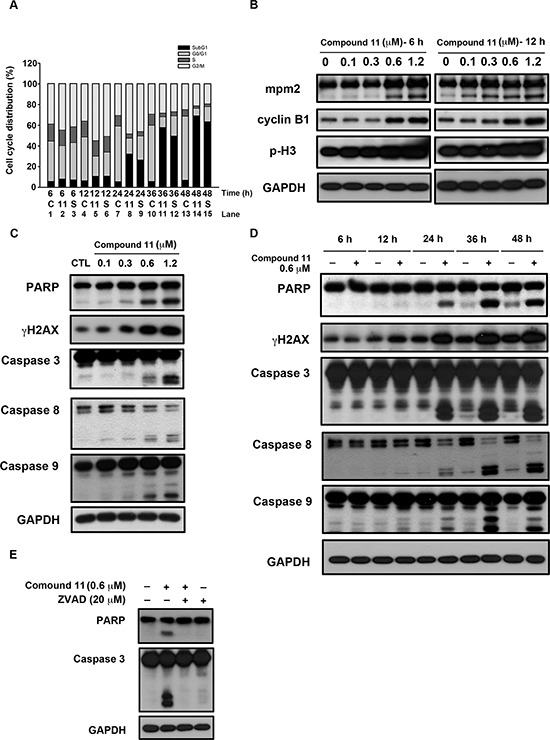
Compound 11 induces cell cycle arrest and apoptotic cell death in HCT116 cells **A.** Effect of compound 11 and SAHA on cell cycle distribution. Cells were treated with compound 11 or SAHA for the indicated times and assessed via flow cytometry. C: control group; 11: compound 11, 0.6 μM; S: SAHA, 1.2 μM. **B.** Concentration-dependent effects of compound 11 on mitotic arrest. Cells were treated with the indicated concentrations of compound 11 for 6 h (left panel) or 12 h (right panel), and cell lysates were subjected to Western blot analysis with the indicated antibodies. p-H3, phosphorylated histone H3 (Ser10). **C–D.** Concentration- (C) and time-dependent effects (D) of compound 11 on apoptotic markers in HCT116 cells. Cells were treated with the indicated concentrations of compound 11 for 48 h or a fixed concentration for different time periods, and whole-cell extracts were subjected to Western blot analysis using the indicated antibodies. **E.** The pan-caspase inhibitor, zVAD, attenuated compound 11-mediated activation of PARP and caspase 3. Cells were exposed to compound 11 in the presence or absence of zVAD for 48 h and extracts subjected to Western blot using the indicated antibodies. zVAD, Z-Val-Ala-Asp(OMe)-FMK.

To establish the mechanism by which compound 11 suppresses cell growth, we initially examined its effect on cell cycle progression via flow cytometry. As shown in Figure 3A, treatment with 0.6 μM compound 11 induced G2/M-phase accumulation at 6–12 h (lane 2 and lane 5) and apoptosis (sub-G1) at 24 hours treatment (lane 8). We noted a consistent increase in the expression levels of general mitotic markers, such as MPM-2, cyclin B1, and phosphorylated histone H3, in drug-treated cells (Figure 3B). Exposure to compound 11 led to a concentration- and time-dependent cleavage of caspase 3, 8, 9 and PARP, and induction of γH2AX in HCT116 cells (Figure 3C and 3D). These data further confirmed the characteristic hypodiploid peak (subG1 phase) that appeared after 24 h of treatment presented in Figure 3A. Furthermore, compound 11-induced apoptosis was prevented upon co-treatment with the pan-caspase inhibitor zVAD (Figure 3E), clearly indicating activation of caspase-dependent cell death in HCT116 cells.

### Effect of compound 11 on Bcl-2 family proteins and survival signaling pathways

**Figure 4:**
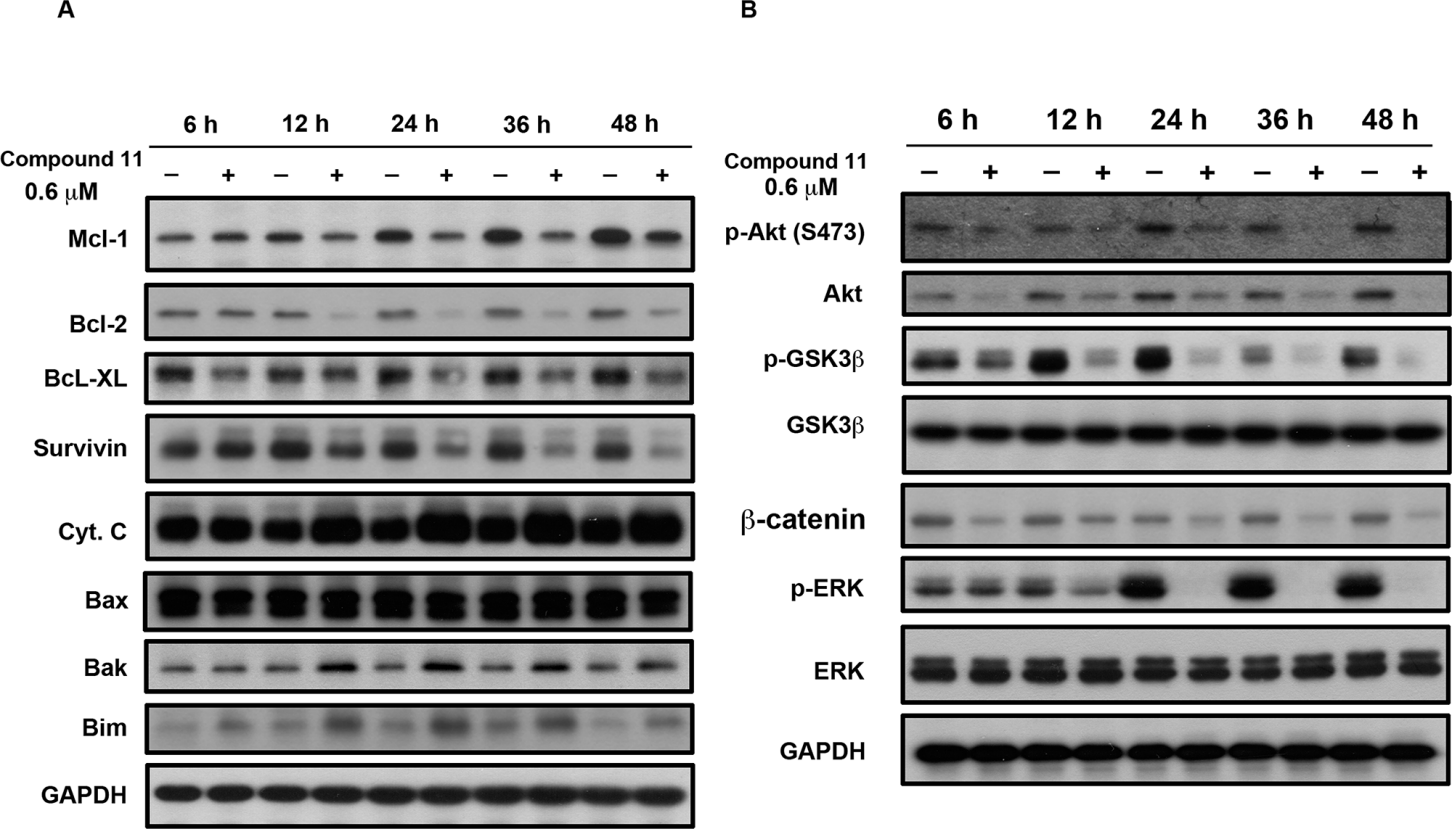
Effects of compound 11 on Bcl-2 family proteins and survival signaling HCT116 cells were exposed to compound 11 (0.6 μM) for the indicated times, and cell lysates were subjected to Western blot analysis using the indicated antibodies against Bcl-2 family proteins **A.** and members of survival signaling pathways **B.** GAPDH servered as a loading control.

Compound 11 induced activation of caspase 3, 8, and 9 in HCT116 cells (Figure 3C and 3D). Caspase 9 and Caspase 8 are indicators of intrinsic mitochondrial and extrinsic membrane apoptotic pathway, respectively. In addition, Bcl-2 family proteins including anti- and pro-apoptotic members, regulate life or death decisions and play important roles in intrinsic apoptotic pathways in cells [13]. In our experiments, the levels of anti-apoptotic proteins, Bcl-2, Mcl-1, survivin, and Bcl-_XL_, decreased in a time-dependent manner following compound 11 treatment (Figure 4A). On the other hand, the proapoptotic proteins Bak, Bim, and cytochrome c were also increased, although the drug had little or no effect on the expression of proapoptotic Bax (Figure 4A). PI3K/Akt and Ras/Raf/MEK/ERK are the major known pathways activated in cancer cells [14–16]. As shown in Figure 4B, treatment with compound 11 dramatically reduced the levels of phospho-Akt (Ser473), and phospho-ERK (Thr202/Tyr204) in HCT116 cells. Notably, protein expression of total Akt was also decreased. The phenomenon was further confirmed by compound 11-mediated suppression of phospho-GSK3β (Ser 9) and its downstream target β-catenin. These data collectively indicate that compound 11 exhibits a potent inhibitory effect on survival signals in HCT116 cells.

### Compound 11 reduced cell migration and reversed the mesenchymal phenotype of HCT116 cells via Akt down-regulation

**Figure 5:**
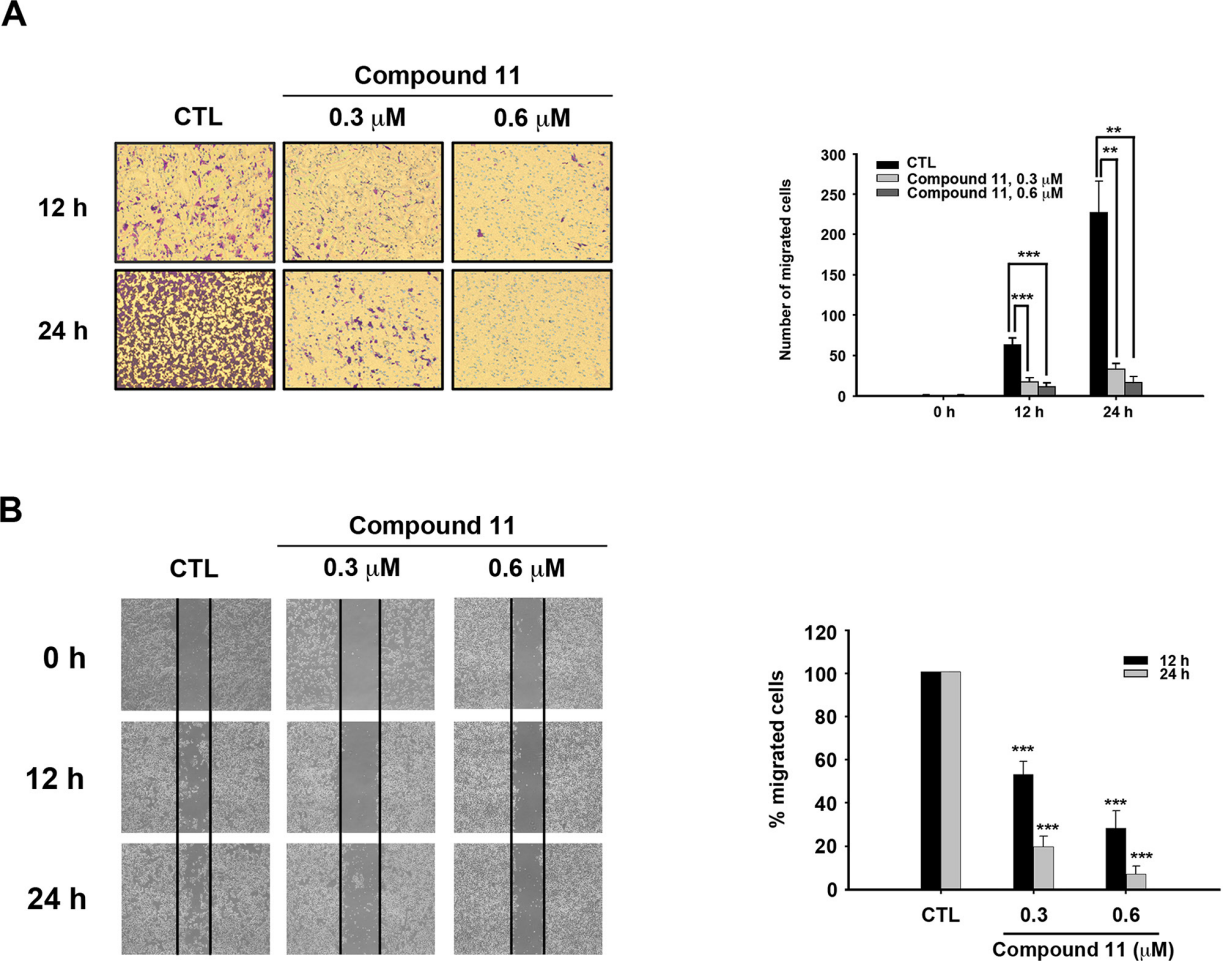
Compound 11 inhibits the migratory ability of HCT116 cells **A.** Cell migration was assessed with the Boyden chamber assay (left panel) in the presence or absence of compound 11 for the indicated times. Numbers of migrated cells are presented on a bar chart (right panel). Data represent the mean ± S.D. from three independent experiments. **P < 0.01; ***P < 0.001, compared with control (CTL) group. **B.** Cells were seeded on 6-well plate and scratches made to allow migration towards the center in the presence or absence of compound 11 for different times periods. The results of the wound healing assay were imaged under microscopy (40 × magnification) after the scratch (left panel). The percentage of migrating cells is shown in a bar chart (right panel). Data represent the means ± S.D. from three independent experiments. **P < 0.01; ***P < 0.001, compared with the control (CTL) group.

**Figure 6:**
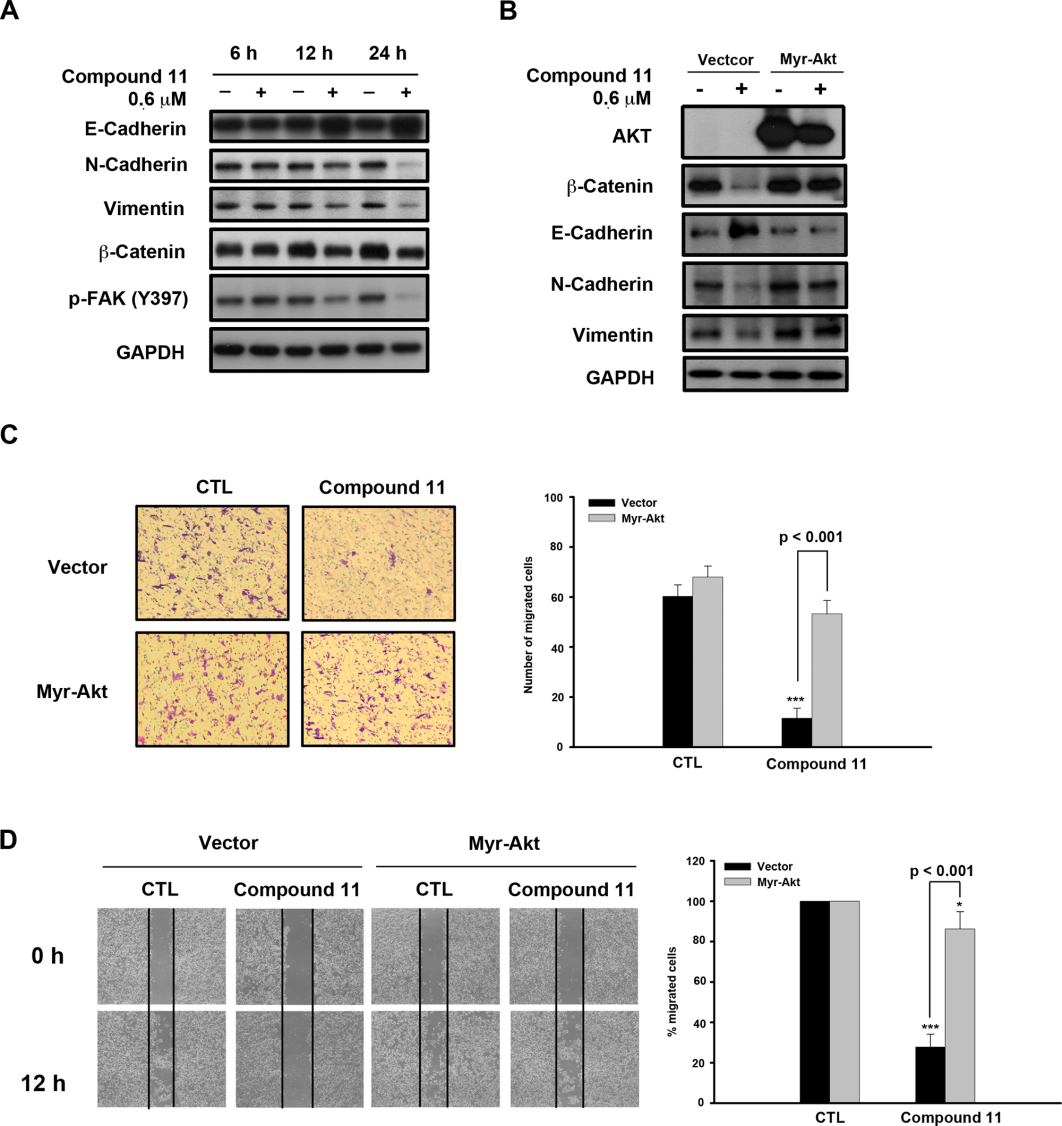
Compound 11 inhibits cell migration through downregulating Akt expression **A.** Effects of compound 11 on expression of EMT markers in HCT116 cells. Cells were exposed to compound 11 for the indicated times, and whole cell lysates subjected to Western blot analysis with the indicated antibodies. **B.** HCT116 cells were transfected with vector or Myr-Akt plasmid for 24 h, and exposed to compound 11 for 48 h. Protein expression of Akt and EMT markers was assessed via Western blot analysis. **C–D.** Vector or Myr-Akt plasmid was ectopically expressed in HCT116 cells, and migratory abilities measured with the Boyden chamber (C) and wound healing assays (D) in the presence or absence of compound 11 (0.6 μM) for 12 h (left panel). Numbers or percentages of migrated cells are presented as mean ± S.D. of three independent experiments (right panel). **P < 0.01; ***P < 0.001 compared with the control (CTL) group.

To establish the effect of compound 11 on metastasis, we investigated whether the drug interferes with cell motility using Boyden chamber assay and wound healing assay. Compound 11 suppressed HCT116 cell migration at non-cytotoxic doses (0.3 μM) to a significant extent (Figure 5A). Consistently, data from the wound healing assay revealed that fewer cells migrated from the wound edge to the center space upon treatment with compound 11 (Figure 5B). Epithelial-mesenchymal transition (EMT) is reported to play a critical role in the invasive and metastatic activity of colorectal cancer [10, 17]. Accordingly, we examined the effects of compound 11 on expression of EMT-associated markers. Compound 11 induced downregulation of mesenchymal markers (N-cadherin and vimentin) and invasive marker (p-FAK), whereas the protein level of the epithelial marker, E-cadherin, was increased (Figure 6A). Given that Akt signaling activation is crucial for EMT in colorectal cancer cells [18, 19] and compound 11 decreases Akt protein expression (Figure 4B), we further examined the putative link between Akt and compound 11-mediated reduction in cell migration and EMT. Ectopically expressed Myr-Akt induced expression of its downstream target β-catenin and rescued the inhibitory effects of compound 11 on EMT-associated proteins (Figure 6B). The finding was further validated by the reversal effects on cell migration, as demonstrated with Boyden chamber (Figure 6C) and wound-healing assays (Figure 6D). Accordingly, these data propose that compound 11 reverses mesenchymal properties and suppresses migration through downregulation of Akt in HCT116 cells.

### Anti-tumor activity of compound 11 in a HCT116 xenograft model

**Figure 7:**
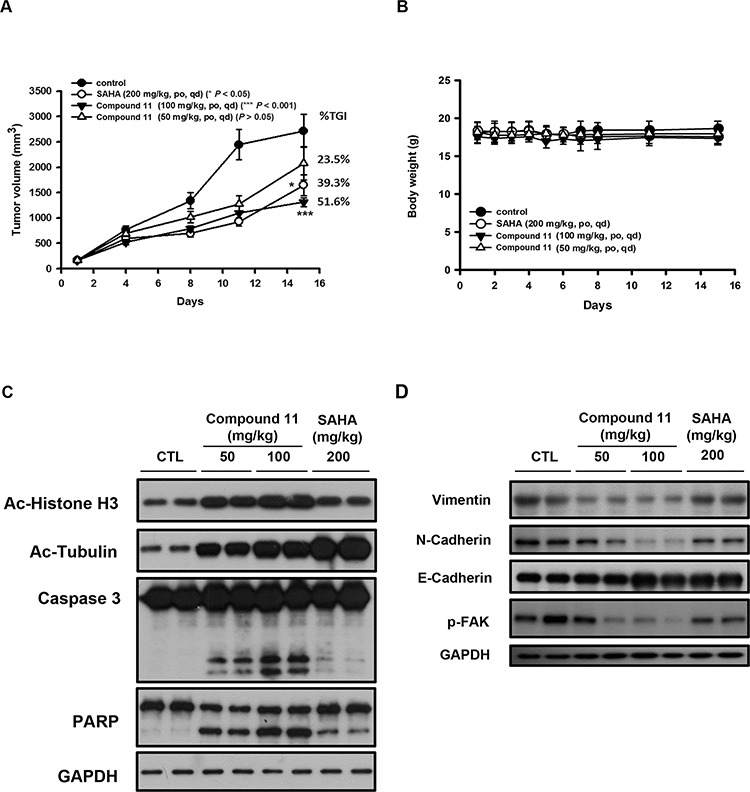
Anti-tumor activity of compound 11 in an HCT116 xenograft model **A.** Athymic nude mice bearing HCT116 tumor xenografts were treated with vehicle or compound 11 (50 or 100 mg/kg) or SAHA (200 mg/kg) per day via oral gavage for 15 days. Eight mice per group were used in the xenograft model, and the tumor volumes of mice were measured. TGI%: percentage of tumor growth inhibition (*P < 0.05; ***P < 0.001). **B.** Body weights of tumor-bearing mice under treatment during the study. Data represent mean ± S.D. from eight mice in each group. **C–D.** Tumor homogenates from two mice from each group were analyzed via Western blot using antibodies against epigenetic/apoptotic markers (C) and EMT markers (D).

To further evaluate the antitumor efficacy of compound 11 in the preclinical setting, athymic nude mice bearing established HCT116 tumor xenografts were treated orally with vehicle or compound 11 at 50 or 100 mg/kg body weight per day. As shown in Figure 7A, oral administration of 50 and 100 mg/kg compound 11 significantly inhibited tumor growth by 23.5% and 51.6%, respectively. Notably, SAHA was less potent than compound 11 with 39.3% tumor growth inhibition (TGI) at 200 mg/kg. In addition, compound 11 was well tolerated by tumor-bearing mice and induced no appreciable change in body weight was observed (Figure 7B). To ascertain whether compound 11-suppressed tumor growth via inhibition of HDACs and induction apoptosis *in vivo*, intratumoral biomarkers were assessed via Western blot. The data showed that compound 11 increases acetylation of histone H3 and α-tubulin as well as the cleavage of caspase-3 and PARP (Figure 7C), consistent with our mechanistic data *in vitro*. Moreover, compound 11 inhibited the expression of mesenchymal markers (N-cadherin and vimentin) and invasive marker (p-FAK), accompanied by the induction of the epithelial marker, E-cadherin (Figure 7D). These results clearly demonstrate that compound 11 induces apoptosis and reverse the mesenchymal phenotype of HCT116 cells *in vivo*.

## DISCUSSION

Colorectal cancer is the third most common malignant neoplasm worldwide and the third highest cause of death in men and women in the United States [20]. Extensive evidence suggests that dysregulation of HDACs is correlated with significant decreases in both disease-free and overall survival in several types of cancer, including colorectal cancer [2]. Among the different HDAC isoforms, expression of class I HDACs has an independent prognostic impact on human colorectal cancer [21]. In the current study, we have provided evidence that compound 11, a derivative of 1-arylsulfonyl-5-(N-hydroxyacrylamide) tetrahydroquinolines, is a novel HDAC inhibitor applicable for the treatment of CRC. Data obtained from a previous *in vitro* HDAC inhibition assay showed that compound 11 is selective for Class I HDACs. Compared with SAHA, compound 11 is 2- to 5-fold more potent against HDAC 1, 2, and 8, but 8-fold less potent in inhibition of HDAC 6 [12]. These results are consistent with our current data that compound 11 induces lower levels of α-tubulin acetylation than SAHA (Figure 2B and 2C). In addition, compound 11 suppressed proliferation and viability in colorectal cancer cell lines, regardless of the mutational status of K-Ras (Figure 1). From a clinical viewpoint, anti-tumor activity of compound 11 in a HCT116 xenograft model was demonstrated *in vivo*, with no adverse effects on body weight. Our results indicate that compound 11 is a potent HDAC inhibitor efficacious for CRC.

The effects of HDAC inhibitors on tumor cells include cell cycle arrest, induction of cell death, senescence, differentiation and autophagy [22, 23]. The ability to cause mitotic arrest reportedly correlates with higher cytotoxicity of HDAC inhibitors [24]. Here, compound 11 induced G2/M cell cycle arrest from 6 to 12 h and pronounced apoptosis after 24 h treatment in a caspase-dependent manner (Figure 3A). The expression levels of mitotic markers, such as MPM-2, cyclin B1, and phosphorylated histone H3, was increased in compound 11-treated cells (Figure 3B). Several kinases and regulatory proteins, such as Aurora B, survivin and RhoA GTPase, are reportedly required for successful cytokinesis [25] Downregulation of Aurora B and survivin proteins via the 26S proteasome pathway upon treatment of HDAC inhibitors has also been reported [26, 27]. Therefore, further investigation of the effects of compound 11 on these enzymes is warranted. We further confirmed that compound 11-induced apoptosis occurs through intrinsic and extrinsic pathways (Figure 3C and 3D). The levels of the anti-apoptotic proteins, Bcl-2, Mcl-1, survivin, and Bcl-XL were decreased, whereas proapoptotic Bak, Bim, and cytochrome c were increased in a time-dependent manner upon compound 11 treatment (Figure 4A). Clearly, compound 11 is able to disrupt cell cycle progression and induce apoptosis in CRC cells.

During embryogenesis of both vertebrates and invertebrates, epithelial cells undergo conversion to a phenotype more amenable to migration and invasion, a process known as the epithelial-mesenchymal transition (EMT). EMT is also a critical process in tumor progression that promotes cell breaching of the basement membrane and metastasis to distant sites [11]. Emerging evidence demonstrates a pathological role of EMT in colorectal cancer that promotes metastasis and resistance to neoadjuvant therapy [28]. Therefore, EMT presents a promising therapeutic target for CRC. Our experiments showed that compound 11 suppresses the migration of CRC cells (Figure 5A and 5B). Compound 11 also increases the epithelial marker E-cadherin, and decreases the mesenchymal markers (N-cadherin and vimentin) as well as the invasive marker (p-FAK) (Figure 6A). Furthermore, these mechanisms may occur through downregulation of Akt by compound 11 (Figure 6B and 6C). HDAC inhibitors are reported to regulate Akt activity directly through modulating gene expression or indirectly through disrupting the interactions between HDAC and protein-phosphatase 1 (PP1) [29]. Valproic acid and butyrate inhibit gene transcription of Akt and cause caspase-dependent apoptosis in cancer cells [30]. In the current study, compound 11 induced a significant decrease in Akt protein expression at 6 h, consistent with the suppression of its downstream targets, pGSK3-β and β-catenin (Figure 4B). The data also demonstrated that both of compound 11 and SAHA decrease the protein expression of Akt and β-catenin in a concentration-dependent manner (Supplementary Figure S1). Given the role Akt in regulating EMT and survival pathways in cancer cells, elucidation of the mechanism underlying compound 11-induced Akt downregulation would be of considerable research interest.

In conclusion, compound 11 is a novel HDAC inhibitor exerting significant anti-tumor activity in CRC cells. Compound 11 induces cell cycle arrest, and activates both intrinsic and extrinsic apoptotic pathways in a caspase-dependent manner. Moreover, compound 11 alters the expression levels of Bcl-2 family proteins and has a potent inhibitory effect on survival signals in CRC cells. Inhibition of cell motility and reversal of the mesenchymal phenotype are induced through downregulation of Akt. Consistent with *in vitro* findings, compound 11 significantly suppresses tumor growth in a HCT116 xenograft model *in vivo*. Our results collectively validate the activity of compound 11 as a novel HDAC inhibitor and support its potential for further development as targeted therapy for CRC.

## MATERIALS AND METHODS

### Cell culture and reagents

Human colorectal cancer HCT116 and HT-29 cell lines were purchased from the American Type Culture Collection. Cells were maintained in 10% fetal bovine serum (FBS)-supplemented RPMI 1640 medium (GIBCO, Grand Island, NY, USA) and 1% penicillin-streptomycin (GIBCO) at 37°C in a humidified incubator containing 5% CO_2_. 3-(4,5-Dimethylthiazol-2-yl)-2,5-diphenyltetrazolium bromide (MTT), propidium iodide (PI), zVAD and all of the other chemical reagents were purchased from Sigma Chemical (St. Louis, MO, USA). Antibodies against various proteins were obtained from the following sources: PARP (Poly-ADP-ribose polymerase), Mcl-1, Bcl-2, Bcl-XL, survivin, cytochrome c, Bax, Bak, Bim, anti-mouse and anti-rabbit IgGs were obtained from Santa Cruz Biotechnology Inc. (Santa Cruz, CA, USA). Phospho-Akt (Ser473), phospho-GSK-3β, Akt, phospho-p44/42 MAPK (1/2 Erk) (Thr202/Tyr204), p44/42 MAPK (1/2 Erk), caspase8, caspase 9, γH2AX, p21 and acetyl-α-tubulin were obtained from Cell signaling (Danvers, MA, USA)., Actin was obtained from Chemicon (Billerica, MA, USA). Acetyl-histone H3 and GPADH were from Millipore (Billerica, MA, USA). Caspase 3 and was obtained from IMGENEX (San Diego, CA, USA).

### Cell viability assay

Cells were seeded in 96-well plastic plates and exposed to DMSO, compound 11, or SAHA for 48 h. Cell viability was assessed using the 3-(4,5-dimethylthiazol-2-yl)-2,5-diphenyltetrazolium bromide assay as described previously [31]. Growth inhibition was expressed as the percentage of surviving cells in drug-treated versus DMSO-treated control cells (which was considered as 100% viability).

### Facscan flow cytometric assay

Cells were seeded in 6-well plates (2.5 × 10^5^/well) and treated with DMSO, compound 11, or SAHA at various concentrations for the indicated times. Cells were washed with phosphate-buffered saline (PBS), fixed in ice-cold 70% ethanol at −20°C overnight, and stained with propidium iodide (80 μg/ml) containing Triton X-100 (0.1%, v/v) and RNase A (100 μg/ml) in PBS. DNA content was analyzed with the FACScan and CellQuest software (Becton Dickinson, Mountain View, CA, USA).

### Western blot and transient transfection

Cells were seeded and allowed to attach overnight. The cells were treated with indicated conditions. After treatment, cells were lysed and the immunoblotting was performed as previous described [31]. For transient transfection, myristoylated Akt (Myr-Akt) plasmid was purchased from Addgene (Cambridge, MA, USA) and transfection was done using lipofectamine 2000 reagent according to the manufacturer's instructions. Following transfection, cells were allowed to recover for 24 h before treatment.

### Cell-based HDAC fluorescence activity assay

HCT116 cells were seeded in 10-cm dish and treated with indicated concentrations of compound 11 and SAHA for 24 h, and then cell lysates were subjected to a HDAC Fluorometric Activity Assay Kit (K330–100, Biovision Inc.) as described previously [32].

### Cell migration assay

Cell migration was performed using 24-well Boyden chamber with 12 μm pore size polycarbonate polyvinylpyrrolidone-free Nucleopore filters (Millipore, Billerica, MA). Briefly, the membrane was coated with 0.5% gelatin for 4 h and cells were trypnized and seeded 100,000 cells/well containing 5% FBS medium in each upper well and added 110 μl 20% FBS medium in each lower chamber. Cells were allowed to migrate in the presence of absence of drugs. After 12 h to 24 h, the membrane was fixed with 4% formalin for 15 min and then stained with 1% crystal violet for 15 min. The cell number was counted in five areas randomly by microscope.

### Wound healing assay

Cells were seeded in 6-well plate to reach 70%–80% confluence, and wound was created by scraping monolayer with a sterile pipette tip. After removal of the cellular debris, the cells were exposed to the indicated drugs for 12 or 24 h. Wound closure was imaged using a light microscope. Traveling distance of the cells was determined by measuring the wound width at 12 h or 24 h, and subtracting it from the wound width at the beginning of treatment (0 h). The values obtained were then expressed as the percent of migration, setting the gap width at 0 h as 100%. Percentages of migrated cells were determined relative to cells treated with DMSO.

### *In vivo* HCT116 xenograft model

Eight-week-old female athymic nude mice were group-housed under conditions of constant photoperiod (12 h light/12 h dark at 21–23°C and 60%–85% humidity) with *ad libitum* access to sterilized food and water. All animal experiments followed ethical standards, and protocols have been reviewed and approved by Animal Use and Management Committee of National Taiwan University (IACUC Approval No: 20100225). Each mouse was inoculated s.c. with 1 × 10^6^ HCT116 cells in a total volume of 0.1 mL serum-free medium containing 50% Matrigel (BD Biosciences). As tumors became established (~100 mm^3^), mice were randomized to four groups (*n* = 8) that received the following treatments: (a) 0.5% carboxymethyl cellulose/0.1%Tween 80 vehicle, (b) Compound 11 at 50 mg/kg/d or (c) 100 mg/kg/d, and (d) SAHA at 200 mg/kg/d. Mice received treatments by gavage for the duration of the study. Tumors were measured weekly using calipers. Tumor size, in mm^3^, was calculated from: where w = width and l = length in mm of the tumor. Tumor volume = (w^2^ × *l*)/2. A portion of each tumor was frozen in liquid nitrogen for Western blotting analysis.

### Statistical analysis

Each experiment was performed at least three times. Data in bar graph are given as the means ± S.D. Means were checked for statistical difference using the *t*-test and *P*-values less than 0.05 were considered significant (*P < 0.05, **P < 0.01, ***P < 0.001).

## SUPPLEMENTARY FIGURE


