# Supplementary material for: Molecular mechanisms underlying the antitumor activity of (E)-N-hydroxy-3-(1-(4-methoxyphenylsulfonyl)-1,2,3,4-tetrahydroquinolin-6-yl)acrylamide in human colorectal cancer cells in vitro and in vivo
Source: Oncotarget. 2015 Oct 5;6(34):35991–6002. doi: 10.18632/oncotarget.5475 (PMC4742156; doi:10.18632/oncotarget.5475)
Supplement: Supplementary file 1 [file oncotarget-06-35991-s001.pdf]

SUPPLEMENTARY FIGURE

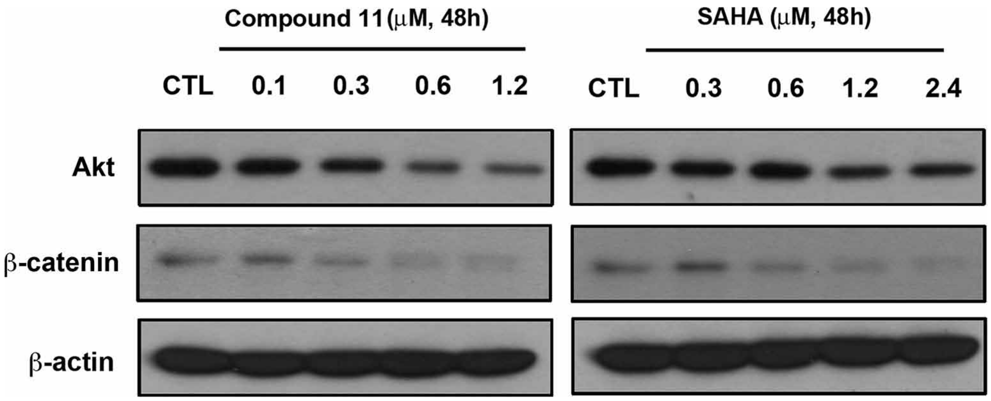

Supplementary Figure S1: Effects of HDAC inhibitors on Akt and β-catenin. HCT-116 cells were treated with indicated concentrations of compound 11 (left panel) and SAHA (right panel) for 48 hours. Protein levels of Akt and β-catenin were detected by western blot analysis.
